# Isometric handgrip exercise impacts only on very short-term blood pressure variability, but not on short-term blood pressure variability in hypertensive individuals: A randomized controlled trial

**DOI:** 10.3389/fphys.2022.962125

**Published:** 2022-09-13

**Authors:** Otávio A. Bertoletti, Rodrigo Ferrari, Elton L. Ferlin, Ozi M. Barcellos, Sandra C. Fuchs

**Affiliations:** ^1^ Programa de Pós-Graduação em Epidemiologia, School of Medicine, Universidade Federal do Rio Grande do Sul, Porto Alegre, Brazil; ^2^ Programa de Pós-Graduação em Cardiologia, School of Medicine, Universidade Federal do Rio Grande do Sul, Porto Alegre, Brazil; ^3^ Serviço de Pesquisa Clínica, Hospital de Clínicas de Porto Alegre, Porto Alegre, Brazil; ^4^ Coordenadoria de Gestão da Tecnologia da Informação e Comunicação, Hospital de Clínicas de Porto Alegre, Porto Alegre, Brazil

**Keywords:** hypertension, blood pressure, blood pressure variability, isometric exercise, handgrip, handgrip dynamometry, average real variability

## Abstract

**Background:** The effect of a single isometric handgrip exercise (IHG) on blood pressure (BP) variability (BPV) has not been addressed. This randomized controlled trial evaluated the effect of IHG vs. sham on BPV and BP.

**Methods:** Hypertensive patients using up to two BP-lowering medications were randomly assigned to IHG (4 × 2 min; 30% of maximal voluntary contraction, MVC, with 1 min rest between sets, unilateral) or sham (protocol; 0.3% of MVC). Systolic and diastolic BP were assessed beat-to-beat in the laboratory before, during, and post-intervention and also using 24-h ambulatory BP monitoring (ABPM). BPV was expressed as average real variability (ARV) and standard deviation (SD).

**Results:** Laboratory BPV, ARV and SD variability, had marked increase during the intervention, but not in the sham group, decreasing in the post-intervention recovery period. The overall change in ARV from pre- to 15 min post-intervention were 0.27 ± 0.07 (IHG) vs. 0.05 ± 0.15 (sham group), with a statistically significant *p*-value for interaction. Similarly, mean systolic BP increased during the intervention (IHG 165.4 ± 4.5 vs. sham 152.4 ± 3.5 mmHg; *p* = 0.02) as did diastolic BP (104.0 ± 2.5 vs. 90.5 ± 1.7 mmHg, respectively; *p* < 0.001) and decreased afterward. However, neither the short-term BPV nor BP assessed by ABPM reached statistically significant differences between groups.

**Conclusion:** A single session of IHG reduces very short-term variability but does not affect short-term variability. IHG promotes PEH in the laboratory, but does not sustain 24-h systolic and diastolic PEH beyond the recovery period.

## Introduction

Poor blood pressure (BP) control increases the risk of cardiovascular disease and mortality (Ettehad et al., 2016), and physical exercise is a sound non-pharmacological strategy to improve wellbeing and BP reduction (Herrod et al., 2018). Current guidelines recommend regular aerobic exercises (alone or combined with dynamic resistance exercises) (Whelton et al., 2018) (Parati et al., 2014) for individuals with hypertension; nevertheless, adherence to an exercise training program is a challenge. In this regard, isometric exercise (Badrov et al., 2016) (Cornelissen and Smart, 2013) may be a compelling alternative, with promising results for BP management (Loaiza-Betancur et al., 2020) (Smart et al., 2019). This type of exercise using a handgrip dynamometer does not depend on sophisticated equipment or a facility and requires a smaller investment of time (Smart et al., 2020) (López-Valenciano et al., 2019); these advantages could help promote adherence for more extended periods.

In addition to reducing BP (Cornelissen and Smart, 2013), the effect of exercise on BP variability (BPV) may contribute to cardiovascular protection because BP fluctuations after each cardiac cycle can predict cardiovascular disease regardless of mean BP levels (Stevens et al., 2016). These BP fluctuations can be determined using different time intervals, from very short (i.e., beat-to-beat) to long-term, visit-to-visit measurements. In addition to the time interval between measurements, the BPV varies with the number of BP measurements (Mena et al., 2014). The most used indices to calculate the BPV are based on the standard deviation (SD) and the coefficient of variation (CV). However, these methods do not assess specific time intervals in which marked variations may occur. The use of 24-h ambulatory BP monitoring (ABPM) to assess BP variations over 24 h (i.e., average real media of BP; ARV) is particularly compelling because it captures BPV modulations during activities of daily living and sleep (Mena et al., 2005), allowing consecutive reading-to-reading measurements; therefore, ABPM could be an option to assess short-term BPV (Del Giorno et al., 2019) (Mena et al., 2017).

Some studies have detected acute variations in blood pressure during (Stewart et al., 2007) or at the recovery period after an IHG session (Millar et al., 2009) (Millar et al., 2011) (van Assche et al., 2017) (Souza et al., 2018), while others have not (Olherdos et al., 2013) (Goessler et al., 2016) (Silva et al., 2018). The so-called post-exercise hypotension (PEH), an acute increase in BP during exercise followed by a reduction at the end of the exercise (Ferrari et al., 2017), could be advantageous if it were able to prolong PEH beyond the recovery period (Carpes et al., 2021). Particularly, the reduction in daytime or even 24-h diastolic BPV, as observed after a single session of beach tennis (Domingues et al., 2022), could be a complementary therapeutic target to be pursued. However, the acute effect of IHG exercise on PEH is controversial (Ash et al., 2017) (Rickson et al., 2021) and poorly explored. There are no studies evaluating the effects of this type of resistance training on BPV using a new index, such as the ARV. The purpose of the present study was to evaluate the effectiveness of a single session of IHG exercise on BPV in hypertensive individuals. As secondary outcomes, daytime, nighttime, 24-h BP, and the safety of this exercise modality were evaluated. The primary hypothesis was that a single session of IHG would decrease BP and its variability compared with a non-exercise control (i.e., sham session).

## Material and methods

### Study design

This study was a randomized, sham-controlled, single-blinded, parallel, superiority clinical trial registered with the Plataforma Brasil (CAAE) database under the registration number 45997915.7.0000.5327. It was approved by the Ethics Committee of Hospital de Clinicas de Porto Alegre (GPPG: 2015-0279), which is an Institutional Review Board accredited by the Office of Human Research Protection, and all patients signed an informed consent form previously to inclusion. The study was conducted in the Clinical Research Center at the Hospital de Clínicas de Porto Alegre (Porto Alegre, RS, Brazil). The protocol followed the Consolidated Standards of Reporting Trials for parallel design trials and all ethical principles of data confidentiality and protection. Recruitment lasted from December 2017 to August 2019, enrolling outpatients and participants through social media and newspaper advertisements.

### Participants

We enrolled individuals aged 30–75 years (men and women) with office BP ≥ 135/85 mmHg and ABPM ≥130/80 mmHg, using up to two antihypertensive drugs and physically inactive (<150 min/week of physical activity). Participants were not eligible if they had a diagnosis of chronic kidney disease, heart failure, diabetes mellitus, current smoking, musculoskeletal problems preventing hand exercising, adequate physical activity (≥150 min/week), and use of hormone replacement therapy.

### Preliminary evaluations

In the first contact, a trained and certified researcher assessed eligibility criteria by telephone. Participants underwent clinical screening during the first visit, including history, standardized office BP, and anthropometric measurements. Office BP assessments were performed in triplicate during the session using an automated BP device (Omron^®^ HEM-705CP; *Omron Matsuzaka*, *Mie*, *Japan*), and the first one was disregarded.

In the second visit, participants underwent office BP measurements and a familiarization session with the handgrip equipment. Standardized instructions of how the equipment works were provided. An extra familiarization session was offered for anyone who needed it. At the end, 24-h BP monitoring was initiated. At the third visit, randomization was performed for those who had confirmed eligibility.

### Randomization and allocation concealment

A randomization list was generated using random blocks of four to six participants (allocation ratio 1:1), stratified by sex and age (30–59 and 60–75 years) to provide homogeneity on prognostic factors. An independent epidemiologist outside the clinical setting generated the randomization list using a website (Randomization.com) and stored it on the RedCap^®^ platform. Participants and the research team were blinded to the randomization list until the assignment.

### Experimental protocols

Participants were randomly allocated to perform a single session of IHG or a time-matched sham exercise. Both interventions were carried out between 7:00 and 11:00 a.m. to control for the cyclical variation in diurnal BP. Each participant was asked not to drink water during the experimental sessions. Continuous and non-invasive BP monitoring was initiated before and during laboratory sessions, with beat-to-beat recording in the non-dominant arm (BIOPAC^®^ Systems, Inc., CNAP^®^ Monitor 500—NIBP100D, CA, United States). BP monitoring was maintained during the recovery period, followed by a 24-h ABPM. The interval between the familiarization and experimental sessions was at least 24 h. The session consisted of 20 min of rest before the experimental sessions and 60 min of recovery.

**FIGURE 1:**
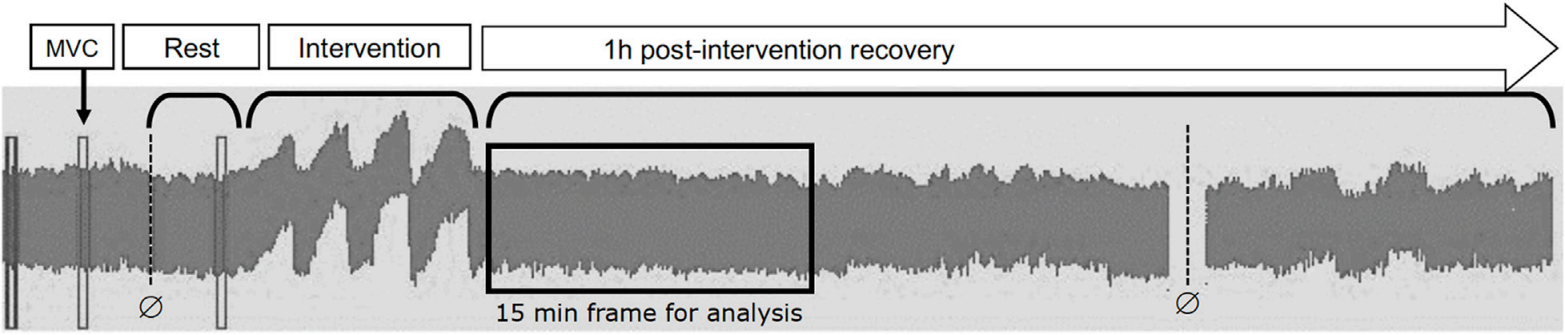
Design of the trial.

Both groups performed a single session of unilateral IHG with an analogue handgrip dynamometer device (JAMAR^®^, Modelo hidráulico 5030J1, *Sammons Preston Rolyan*, *Bolingbrook*, *IL*, *USA*) with a precision of 0.5 kgf, performed on the non-dominant hand. The maximal voluntary contraction (MVC) was established on the day of the intervention, with three attempts of maximum effort and a 1-min rest between trials (Figure 1). During the testing, participants were verbally encouraged to maintain the handgrip contraction, and the highest value obtained was used as a reference to perform the IHG session.

IHG participants performed four sets of 2 minutes each, with a 1-min rest between sets, totaling approximately 12 min of exercise. During each set, participants were asked to maintain approximately 30% of MVC, and verbal feedback was provided during the exercise to maintain the intensity of the handgrip. Participants remained seated, feet completely flat on the floor, the back and forearm supported on the back and arm of the chair (respectively), the wrist in the neutral and free support position, the elbow flexed at 90°, and the shoulder slightly adducted and in the neutral position. Participants allocated to the sham group maintained the same exposure time (face time) during the session. They performed the same assessment procedures, following a similar protocol (i.e., four sets of 2 minutes each), except for the exercise intensity, which was 0.1 kgf, corresponding to an average of 0.3% of the MVC.

### Outcome measurements

ABPM was performed every 15 min during the daytime (starting after the experimental session until the nighttime period and continuing until 11 a.m.) and 20 min during the nighttime period (11 p.m.–7 a.m.). Participants completed a diary recording the type and time of activities, symptoms, sleeping, and awakening. The ABPM was considered valid when there were recordings of at least 14 and 7 daytime and nighttime readings, respectively. All valid BP records obtained using ABPM were exported to an Excel worksheet and opened in a Microsoft Excel (2013) worksheet developed to calculate the ARV. The weighted ARV for the interval between consecutive readings was calculated for systolic and diastolic BP during the daytime, nighttime, and 24-h periods. The ARV index represents the absolute difference between two consecutive measures to demonstrate the true reading-to-reading variability (Mena et al., 2005). A quality control procedure was performed before each new assessment, with an automatic calibration process to parameterize the BP measurements through the brachial cuff and cuffs placed on the proximal phalanges of the index and middle fingers of the same upper limb. The BP captured by the continuous beat-to-beat monitoring system was analyzed during the first 5, 10, and 15 min of the recovery phase after the interventions. The very short-term BPV was calculated using software developed in Visual Basic for Applications especially for this study, for BP data captured by the continuous beat-to-beat monitoring system in the laboratory, allowing reconstruction, filtering, and analysis of the pressure wave during the pre- and post-intervention phases. BP data were analyzed for the first 15 min before starting the automatic recalibration during the recovery phase.

### Statistical analysis

The sample size was estimated using EPIDAT (PHARO, version 4.2), according to the results of a study that evaluated the effect of a single bout of resistance exercises, which apply the maximum amount of force as fast as possible, on BP assessed by ABPM in hypertensive individuals as done in our study (Carpes et al., 2021). The sample size estimated that 72 individuals with hypertension (36 in each experimental session) would be able to detect a difference of 6 mmHg in systolic BP between the protocols with 80% statistical power and a type I error <0.05. At the end of the trial, we did a post-hoc power calculation for systolic BPV for the change in ARV from pre- to 15 min post-intervention comparing the IHG and sham group. Data were entered in the RedCap^®^ platform with quality control and exported to the SPSS Statistics for Windows, version 23.0 (IBM, Armonk, NY). Results were expressed as means ± SD or mean ± SE for variables with normal distribution or medians and interquartile range for non-normal distributions and 95% confidence intervals (95% CI). Generalized Estimating Equations analysis was used to compare the main effects between experimental groups using systolic and diastolic BPV and variability for 24-h BP values, assessing the condition (isometric and sham exercise) by time (three factors: daytime, nighttime, and 24-h). In the laboratory, beat-to-beat BP values have been obtained for each time period and BPV indices were calculated. Therefore, the GEE analysis included pre-, session, and 5, 10 and 15 min post-intervention BPV, eg comparing the ARV index between the IHG and sham group. Post-hoc comparisons were performed using the Bonferroni test. Statistical significance was set at *p* < 0.05.

## Results

**FIGURE 2:**
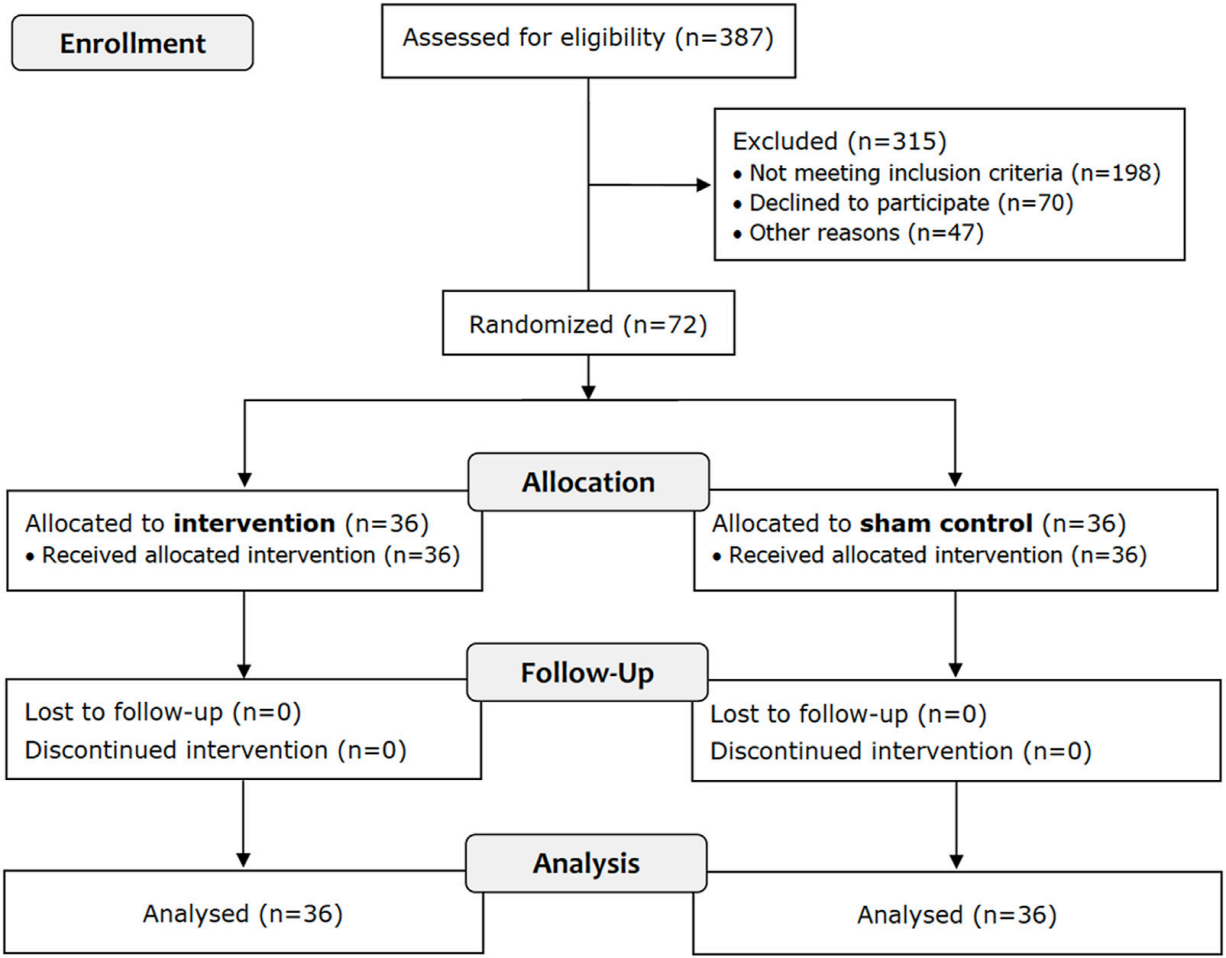
Flow chart of the trial.

A flowchart of the trial is presented in Figure 2, showing that 72 of 387 participants met the eligibility criteria, and all completed the trial.

Table 1 shows that participants had approximately similar characteristics and were (on average) overweight, had increased office BP, and most were taking one BP-lowering drug. There were no reported adverse events during the experimental and sham sessions.

**TABLE 1:** Characteristics of the participants at the baseline according to the allocation group.

	Isometric exercise *n* = 36	*Sham n* = 36
Men	18 (50.0)	17 (47.2)
Age (years)	56.9 ± 10.7	56.4 ± 10.3
Years completed at school	12.2 ± 3.0	12.6 ± 5.8
Body mass index (kg/m^2^)	29.3 ± 4.5	29.0 ± 5.4
Duration of hypertension (years)		
<5	17 (47.2)	14 (38.9)^Ұ^
≥5	19 (52.8)	21 (58.3)
Number of BP lowering drugs		
None	5 (13.9)	1 (2.8)
1	20 (55.6)	21 (58.3)
2	11 (30.5)	14 (38.9)
Blood pressure lowering drug classes		
Diuretics	17 (47.2)	17 (47.2)
Angiotensin-converting enzyme inhibitors	13 (36.1)	9 (25.0)
Angiotensin II receptor blockers	12 (33.3)	17 (47.2)
Calcium channel blockers	2 (5.6)	9 (25.0)
Beta blockers	2 (5.6)	2 (5.6)
Office Systolic BP (mmHg)	142.4 ± 15.6	147.1 ± 16.3
Office Diastolic BP (mmHg)	88.4 ± 12.3	91.5 ± 8.6

Values expressed as *n* (%) or mean ± standard deviation. ^Ұ^ Missing data from one participant. BP: blood pressure.

Table 2 shows the very short-term variability between IHG and sham groups during the experimental sessions at the laboratory. In the IHG group, ARV and SD increased during the intervention with IGH, but not in the sham group. BPV decreased in the post-intervention recovery period and the overall difference in ARV from pre- to 15 min post-intervention was 0.27 ± 0.07 in the IHG vs. 0.05 ± 0.15 in the sham group, with a statistically significant *p*-value for interaction. This post-hoc analysis had 100% statistical power.

**TABLE 2:** Very short-term BP variability assessed at the laboratory before, during and after the isometric handgrip exercise (IHG) and sham sessions.

	IHG exercise (*n* = 34)	Sham (*n* = 33)	*p* value for time	*p* value for group	*p* value for interaction
ARV for Systolic BP (mmHg)			0.030	0.755	0.036
Baseline	1.74 ± 0.53^*^	2.08 ± 0.82			
Session	2.40 ± 0.91^a^ ^,^ ^d^	2.11 ± 0.78			
Recovery period (min)					
5	2.09 ± 0.74^a^	2.08 ± 0.61			
10	2.06 ± 0.83^a^	2.11 ± 0.57			
11–15	2.01 ± 0.71^a^	2.13 ± 0.60			
ARV for Diastolic BP (mmHg)			0.003	0.366	<0.001
Baseline	1.29 ± 0.36^*^	1.60 ± 0.54			
Session	1.86 ± 0.52^*^ ^a^ ^,^ ^b^ ^,^ ^c^ ^,^ ^d^	1.53 ± 0.38			
Recovery period (min)					
5	1.50 ± 0.50^a^	1.50 ± 0.44			
10	1.46 ± 0.59^a^	1.64 ± 0.35			
11–15	1.45 ± 0.46^*^ ^a^	1.67 ± 0.38			
SD variability for Systolic BP (mmHg)			<0.001	<0.001	<0.001
Baseline	3.61 ± 1.15^*^	4.21 ± 1.33			
Session	11.91 ± 4.91^*^ ^a^ ^,^ ^b^ ^,^ ^c^ ^,^ ^d^	4.39 ± 1.68			
Recovery period (min)					
5	5.54 ± 2.19^*^ ^a^	3.84 ± 1.03			
10	4.51 ± 1.42^a^ ^,c^	4.43 ± 1.39			
11–15	4.43 ± 1.67^a^ ^,^ ^b^ ^,^ ^c^	4.42 ± 1.60			
SD variability for Diastolic BP (mmHg)			<0.001	<0.001	<0.001
Baseline	3.02 ± 0.99	3.48 ± 1.04			
Session	11.78 ± 5.23^*^ ^a^ ^,^ ^b^ ^,^ ^c^ ^,^ ^d^	3.66 ± 1.02			
Recovery period (min)	3.74 ± 1.25^a^	3.36 ± 0.88			
5	3.33 ± 1.21	3.81 ± 1.03			
10	3.49 ± 1.21^a^	3.92 ± 1.03			
11–15	3.02 ± 0.99	3.48 ± 1.04			

Values expressed as mean ± standard deviation ARV: average real variability; SD: standard deviation variability *p* values for between group analysis: *p < 0.05 between groups.

a *p* < 0.05 vs. baseline.

b *p* < 0.05 vs. 5 min recovery period.

c *p* < 0.05 vs. 10 min recovery period.

d *p* < 0.05 vs. 15 min recovery period.

The short-term variability after the experimental session is presented in Table 3. There were no statistically significant differences between the IHG and sham groups for ARV and SD variability assessed by 24-h, daytime, and nighttime systolic and diastolic BP.

**TABLE 3:** Short-term variability assessed through ARV and SD for Systolic and Diastolic blood pressure obtained at the 24-h ABPM.

	Isometric exercise (*n* = 36)		*Sham* (*n* = 36)		*p* value for interação
	Before	After	Before	After	
Systolic BP variability (mmHg)					
24-h ARV	11.5 ± 2.9	11.8 ± 2.7	12.0 ± 2.6	11.9 ± 3.1	0.569
Daytime ARV	11.7 ± 3.9	11.9 ± 2.9	11.9 ± 2.8	11.9 ± 3.4	0.733
Nighttime ARV	11.7 ± 3.3	11.5 ± 3.5	11.9 ± 3.5	11.8 ± 3.4	0.967
24-h SD	17.4 ± 4.4	16.7 ± 3.3	17.2 ± 3.4	16.9 ± 3.7	0.651
Daytime SD	14.9 ± 4.7	14.8 ± 4.1	14.8 ± 3.5	14.7 ± 3.9	0.962
Nighttime SD	13.2 ± 4.0	13.0 ± 3.4	12.7 ± 3.6	13.3 ± 4.0	0.421
Diastolic BP variability (mmHg)					
24-h ARV	6.6 ± 1.7	6.8 ± 1.9	7.4 ± 2.4	7.1 ± 2.2	0.174
Daytime ARV	6.9 ± 2.6	7.1 ± 2.3	7.6 ± 2.8	7.5 ± 3.0	0.463
Nighttime ARV	6.5 ± 2.0	6.1 ± 1.9	6.7 ± 2.4	6.1 ± 1.8	0.721
24-h SD	10.1 ± 2.0	9.9 ± 2.1	10.3 ± 2.4	10.1 ± 2.6	0.879
Daytime SD	8.3 ± 2.2	8.7 ± 2.5	9.0 ± 2.9	8.9 ± 2.9	0.404
Nighttime SD	7.7 ± 2.0	7.3 ± 2.6	7.5 ± 2.3	7.6 ± 2.3	0.571

Values are mean ± standard deviation. ARV: average real variability; SD: standard deviation variability.

**FIGURE 3:**
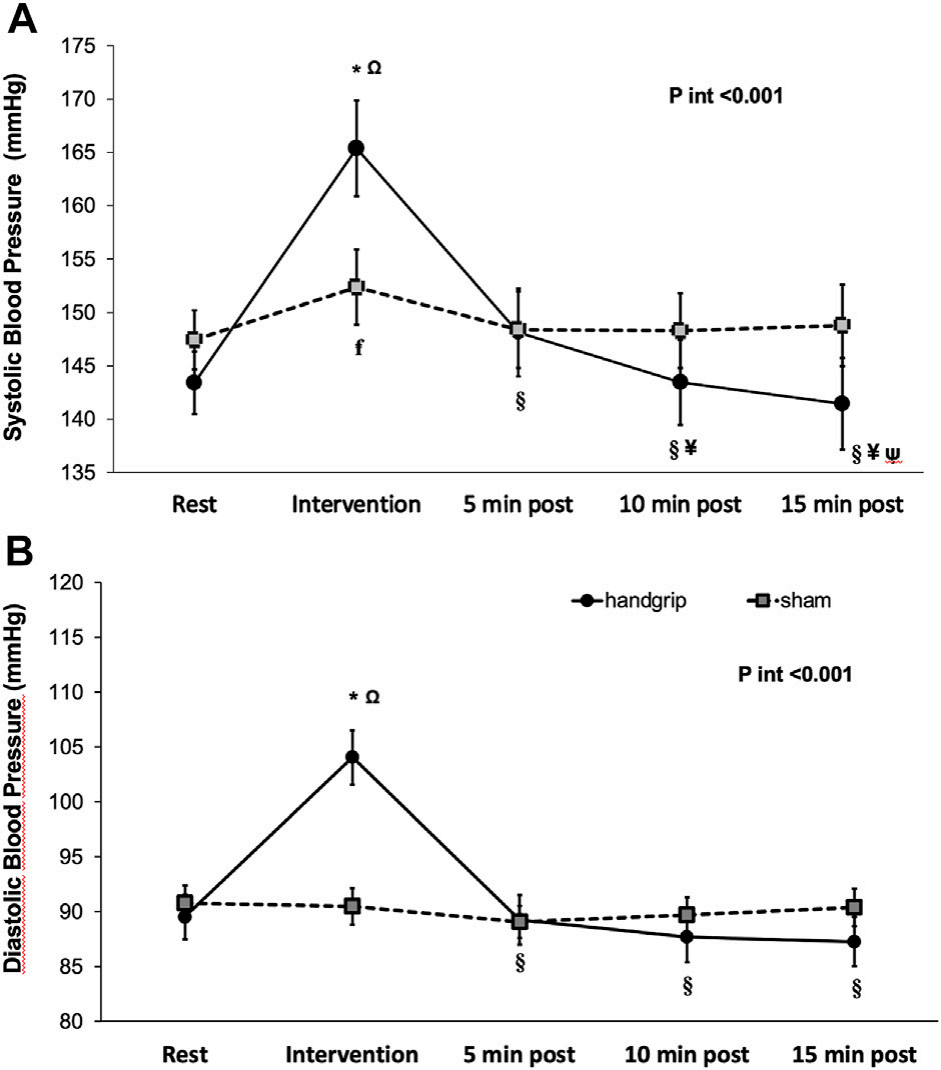
Systolic blood pressure **(A)** and Diastolic blood pressure **(B)** at laboratory during baseline, session and recovery period (mean ± SE). Legend: *P*_*int*_ *, p* value for interaction *p < 0.03 *between groups*; ^Ω^ *p* < 0.001 vs. baseline ^ꞙ^ *p* < 0.05 vs. baseline ^§^ *p* < 0.001 vs. session; ^#^ *p* < 0.05 vs. session; ^¥^ *p* < 0.05 vs.5* *min recovery period; ^ψ^ *p* < 0.05 vs. 10* *min recovery period.

Figure 3 shows the change in BP at the laboratory from the pre- to 15 min post-intervention recovery period within and between groups. Systolic BP increased during the intervention (IHG 165.4 ± 4.5 vs. sham 152.4 ± 3.5 mmHg) as did diastolic BP (104.0 ± 2.5 *v*s. 90.5 ± 1.7 mmHg, respectively) and decreased afterward. There was a statistically significant interaction between group*time*PEH for both systolic and diastolic BP.

In addition, there were no marked differences in systolic and diastolic BP evaluated by ABPM for 24-h, daytime, and nighttime BP between IHG and sham group.

## Discussion

We conducted a randomized controlled trial with a parallel design to test primarily the efficacy of a single session of IHG on short-term BPV and BP, assessed by ABPM. Reduction of systolic and diastolic BPV after the IHG intervention were observed at the laboratory, but no differences between the experimental sessions were found for 24-h, daytime, or nighttime systolic and diastolic BPV. In contrast to our working hypothesis, a single bout of IHG impacted the very short-term BPV, but did not on short-term variability assessed by ARV and SD methods. The result suggests that the BPV reduction in the recovery period was not prolonged during the subsequent 24-h period. There are few studies evaluating the effect of exercise and short-term BPV (Domingues et al., 2022) (Ash et al., 2017) (Carpes et al., 2021); however, none assessed the effect of IHG. Our findings at the laboratory suggest that a single bout of isometric resistance exercise with handgrip (at 30% CVM) was sufficient to acutely reduce resting systolic and diastolic BP, but not to sustain PEH afterward, as it has been shown (Ash et al., 2017). The lack of effect on 24-h BP was also confirmed in resistance (Queiroz et al., 2015) and other types of exercises (Saco-Ledo et al., 2021). A session of recreational sport was able to decrease short-term diastolic BPV (Domingues et al., 2022) and systolic and diastolic BP by 24-h, daytime and nighttime (Carpes et al., 2021).

In addition to reducing BP levels, a reduction in BP variability might contribute to cardiovascular protection (Mena et al., 2017), and it is critical to determine whether an acute exercise session can modulate short-term BP variability (Rickson et al., 2021). The mechanisms that associate BP variability with cardiovascular events are not fully understood, and short- and long-term PEH reflect different physiological and pathological phenomena (Saco-Ledo et al., 2021) (Juhanoja et al., 2016) of BPV (Mena et al., 2017) (Domingues et al., 2022) (Diaz et al., 2014). Therefore, the clinical relevance of each BPV measurement depends on the method and the interval between measurements because different mechanisms and extrinsic factors can determine BP fluctuations (Juhanoja et al., 2016). In particular, the short-term BPV (the subject of the present study) is also modulated by environmental, behavioral, emotional, and postural factors affecting the physiology of the cardiovascular system (Diaz et al., 2014). BPV is a physiological marker of control of the autonomic nervous system because higher fluctuations in BP may be associated with damage to the autonomic system (Queiroz et al., 2015). In addition, studies performed on rats demonstrated that high beat-to-beat variability was associated with the development of endothelial dysfunction and atherosclerosis (Sasaki et al., 1994).

Our study has strengths, including using a standardized exercise protocol that is time-efficient, does not rely on expensive equipment, and performs ABPM assessment. However, some limitations must be considered when interpreting the results. The analogue handgrip equipment did not have a visual mechanism to provide feedback to the participant to maintain constant effort and intensity during the isometric exercise. Nevertheless, we believe that potential fluctuations were minimized by maintaining continuous verbal feedback during the experimental period. Additionally, the enrollment of untrained participants may have affected our findings’ generalization to other populations. Finally, our sample size estimate was based on BP results from another type of exercise performed in a sample of elderly individuals with hypertension, assessed by ABPM. Despite of this drawback, the post-hoc power calculation for ARV for systolic BP showed that we carried out the analysis with full statistical power. In conclusion, a single session of IHG does not reduce short-term BP variability and 24-h BP, but the very short-term variability, in adults with hypertension on blood pressure lowering drugs. The present results suggest the need for further studies to assess the potential efficacy of isometric handgrip exercise in reducing ABPM and its variability acutely.

## Data Availability

The raw data supporting the conclusion of this article will be made available by the authors without undue reservation.

## Abbreviations

BP, blood pressure; ARV, average real variability; SD, standard deviation; ABPM, ambulatory blood pressure monitoring.
